# Simultaneous voltammetric sensing of Zn^2+^, Cd^2+^, and Pb^2+^ using an electrodeposited Bi–Sb nanocomposite modified carbon paste electrode†

**DOI:** 10.1039/d3ra00168g

**Published:** 2023-03-02

**Authors:** E. A. Shalaby, A. M. Beltagi, A. A. Hathoot, M. Abdel Azzem

**Affiliations:** a Electrochemistry Laboratory, Chemistry Department, Faculty of Science, Menoufia University Shebin El-Kom 32511 Egypt optimisticessam@yahoo.com; b Department of Chemistry, Faculty of Science, Kafrelsheikh University Kafrelsheikh 33516 Egypt

## Abstract

A sensor for detecting Zn^2+^, Cd^2+^, and Pb^2+^ ions simultaneously based on the square wave anodic stripping response at a bismuth antimony (Bi–Sb) nanocomposite electrode was developed. The electrode was prepared *in situ* by electrodepositing bismuth and antimony on the surface of a carbon-paste electrode (CPE) while also reducing the analyte metal ions. The structure and performance of the Bi–Sb/CPE electrode were studied using scanning electron microscopy, X-ray diffraction, electrochemical impedance spectroscopy, and cyclic voltammetry. Operational conditions including the concentration of Sb and Bi, the type of electrolyte, pH, and preconcentration conditions were optimized. The linear ranges were determined to be 5–200 μg L^−1^ for Zn^2+^, 1–200 μg L^−1^ for Cd^2+^, and 1–150 μg L^−1^ for Pb^2+^ with the optimized parameters. The limits of detection were 1.46 μg L^−1^, 0.27 μg L^−1^, and 0.29 μg L^−1^ for Zn^2+^, Cd^2+^, and Pb^2+^, respectively. Furthermore, the Bi–Sb/CPE sensor is capable of selective determination of the target metals in the presence of the common cationic and anionic interfering species (Na^+^, K^+^, Ca^2+^, Mg^2+^, Fe^3+^, Mn^2+^, Co^2+^, Cl^−^, SO_4_^2−^ and HCO_3_^−^). Finally, the sensor was successfully applied to the simultaneous determination of Zn^2+^, Cd^2+^, and Pb^2+^ in a variety of real-world water samples.

## Abstract 2

A highly sensitive sensor. A sensor for detecting Zn^2+^, Cd^2+^, and Pb^2+^ ions simultaneously based on the square wave anodic stripping response at a bismuth antimony (Bi–Sb) nanocomposite electrode was developed.

## Graphical abstract

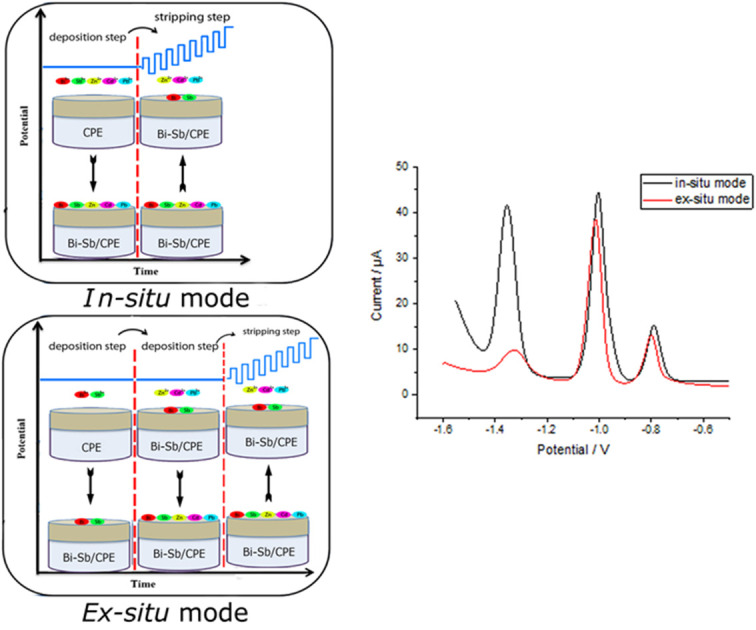

## 1 Introduction

Heavy metals are highly persistent pollutants^1^ and their levels in the environment have risen dramatically in recent decades, possibly as a result of increased industrial and mining activities. The presence of heavy metals, like lead, cadmium and zinc, in water supplies poses a major health hazard.^2^ Lead and cadmium can cause a variety of cancers^3^ as well as serious brain and kidney damage.^4^ Excess zinc, despite its numerous essential biological roles, causes anosmia by rendering the liver or kidneys ineffective.^5^

Numerous analytical methods for determining trace amounts of hazardous heavy metal ions (HMIs) have been developed over the last few decades. These include atomic fluorescence spectrometry,^6^ atomic emission spectrometry, atomic absorption spectrometry,^7^ colorimetry,^8,9^ inductively coupled plasma atomic emission spectrometry, and inductively coupled plasma mass spectrometry.^10,11^ However, while these techniques are very selective and sensitive, they present a number of drawbacks in the detection of HMIs, including high cost, complex operating procedures, and poor field applicability.^12^

Electrochemical techniques, such as stripping voltammetry provide an attractive methodology for detecting HMIs, offering advantages for multiplexed detection in terms of accuracy, sensitivity, simplicity, low cost, and suitability for on-site detection.^13–15^ Anodic stripping voltammetry (ASV) is based on a two-step process. The first step is to pre-concentrate or electrodeposite the heavy metal at the electrode surface by reducing the metal ions. The second step is stripping, which involves re-oxidation of the investigated metals back to produce the ions in solution, according to the following equations:^16,17^M^*n*+^ + *n*e^−^ → M (deposition step)M → M^*n*+^ + *n*e^−^ (stripping step)

Due to ready alloy formation between mercury and HMIs, mercury electrodes have been employed to detect HMIs with good sensitivity and reproducibility. However, they can cause harm to human health and the environment.^18^ The first application of bismuth film electrodes (BiFEs) was reported in 2000,^19^ and since then, the entire topic has already attained a respectable position within the research activities focused on the development of mercury-free electrodes. The uses of BiFEs in stripping voltammetry and stripping chronopotentiometry of metal ions, particularly cadmium, lead, zinc, indium, and thallium, have drawn the interest.^20,21^ Antimony-based film electrodes (SbFEs) prepared *in situ* on the glassy carbon or carbon paste substrate electrode revealed low background currents and a favorable negative overvoltage for hydrogen evolution, similarly as encountered in the case of its bismuth and mercury analogues. The SbFEs also demonstrated a distinguishing characteristic linked to an extraordinarily low antimony oxidation signal, which allowed the detection of analytes with their oxidation potentials close to antimony's oxidation potential.^22,23^ Moreover, the presence of Sb in the electrode material can enhance the catalytic effect in determination of some HMIs such as Cd and Zn due to the formation of CdSb^24^ and ZnSb^25^ intermetallic compounds.

In recent years, nanoparticles, particularly metal nanoparticles, have shown various advantages in the field of electrochemical sensing.^26,27^ Because of their small dimensions, they greatly enhance electrode surface areas. Furthermore, metallic nanoparticles are able to improve the sensitivity of electrodes by providing fast electron transfer and increasing the mass-transport rate.^28^ Bismuth nanoparticles (BiNPs) have been widely employed to modify the surfaces of a variety of electrodes,^29–32^ while the application of antimony nanoparticles (SbNPs) is a new approach to modifying working electrodes for the detection of heavy metals.^33–35^ Accordingly, the development of a bimetallic Bi–Sb-modified electrode would combine the advantages of bismuth and antimony, improving sensitivity and affinity for several metals with low detection limits.

In this work, an electro-sensor was prepared *in situ* by electrodepositing bismuth and antimony on the surface of a carbon-paste electrode (Bi–Si/CPE). Then, its square wave anodic stripping voltammetry (SW-ASV) response was used to simultaneously determine Zn^2+^, Cd^2+^, and Pb^2+^ in water samples.

## 2 Experimental

### 2.1 Materials

Standard stock solutions (1 g L^−1^ Zn^2+^, Cd^2+^, Pb^2+^, Bi^3+^, and Sb^3+^) were supplied by Merck. Acetic acid (CH_3_COOH), sodium acetate (CH_3_COONa·3H_2_O), graphite powder, and Nujol oil were obtained from Sigma Aldrich. Freshly bi-distilled water was used. The real water samples were: tap water; Aquafina® bottled water; underground water from a well located in Shebin Elkom City, El-Menoufia Governorate, Egypt; and treated waste water from a treatment station located in Quesna City, El-Menoufia Governorate, Egypt.

### 2.2 Instruments

Epsilon-EC voltammetric analyzer device from BASi was used to conduct cyclic voltammetry (CV) and SW-ASV analyses. The three-electrode system consisted of a holder electrode with diameter 3.0 mm filled with carbon paste (graphite and Nujol oil), a silver/silver chloride (Ag/AgCl) reference electrode, and an auxiliary electrode made of platinum (Pt) wire. A Model Versa STAT 4 potentiostat (Princeton Applied Research, Oak Ridge, TN, USA) was used to conduct electrochemical impedance spectroscopy (EIS) experiments. EIS was fitted by the equivalent circuit modeling applying the ZSim ver. 3.20 software (EChem Software, Michigan, USA). Scanning electron microscopy (SEM) images and energy-dispersive X-ray (EDX) analysis were achieved using a Thermo Scientific Quattro (Netherlands). The X-ray diffraction (XRD) patterns were carried out by Shimadzu 6000, the measurement conditions used were Cu target radiation, voltage 40.0 kV, and current 30.0 mA at room temperature.

### 2.3 Modified-carbon-paste electrode preparation

**Fig. 1:**
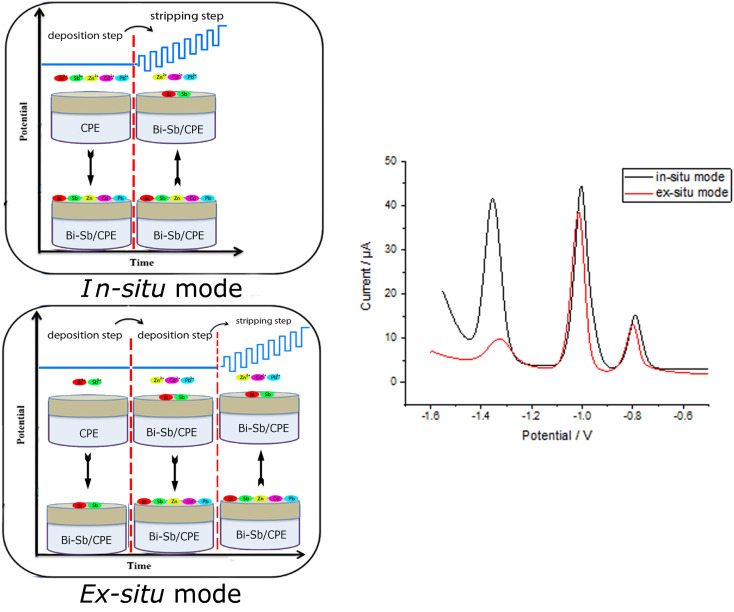
Schematic representation of *in situ* and *ex situ* methods used for preparation of the Bi–Sb/CPE modified electrode and for determination of Zn^2+^, Cd^2+^, and Pb^2+^ metal ions.

In a small mortar, 5 g graphite powder (1–2 μm) and 1.8 mL Nujol oil (*d* = 0.84 g mL^−1^) were thoroughly milled to make a homogeneous carbon paste. Carbon paste was then packed into the electrode body and its surface was gently smoothed until shiny appearance. The bismuth-antimony (Bi–Sb) nanocomposite was electrodeposited on the CPE surface from a solution of acetate buffer (pH 5.6) containing 100 μg L^−1^ of each of Bi^3+^ and Sb^3+^ ions only (*ex situ*) or together with the target metal ions (Zn^2+^, Cd^2+^, and Pb^2+^) (*in situ*) as shown in Fig. 1. Electrodeposition was carried out under mild stirring conditions (at 400 rpm) at a deposition potential (dp *E*) = −1.55 V and deposition time (dp *t*) = 180 s. The modified Bi–Sb/CPE was washed by double distilled water and then keep to dry at room temperature prior to use. All experimental work was employed in room temperature.

### 2.4 Procedure for the determination of Zn^2+^, Cd^2+^, and Pb^2+^

The prepared CPE was placed in a solution of acetate buffer (pH 5.6). To the buffer solution, standard HMI solutions (Zn^2+^, Cd^2+^, Pb^2+^, Bi^3+^, and Sb^3+^) were added. SW-ASV was performed in two steps. Firstly, metal deposition at deposition potential (dp *E*) = −1.55 V and deposition time (dp *t*) = 180 s, followed by stripping performed using SWV in a potential range from −1.55 to −0.5 V at a frequency of 15 Hz, potential steps of 6 mV, and a pulse amplitude of 30 mV.

## 3 Results and discussion

### 3.1 SEM, EDX and XRD characterization of different electrodes

**Fig. 2:**
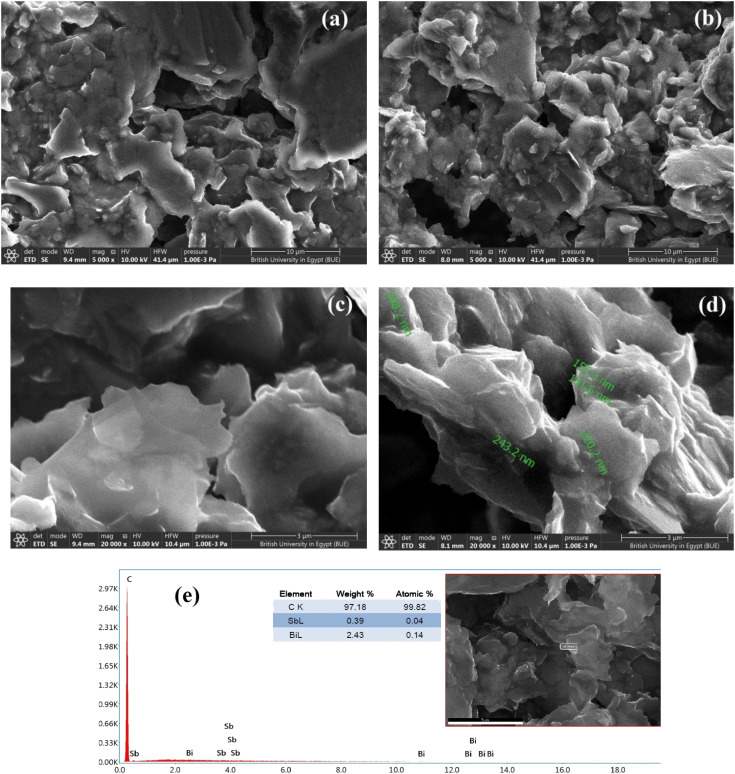
SEM image of bare CPE (a, c) with magnification degrees = 5000× and 2000×, respectively, modified Bi–Sb/CPE (b, d) with the same two magnification degrees and EDX of the modified electrode surface (e).

SEM was used to characterize the morphologies of the electrodes. The SEM micrographs show the morphologies of the deposited SbNPs and BiNPs on the surface of the carbon paste at different magnifications to clarify the details of the surface (Fig. 2a–d). Furthermore, EDX analysis was used to identify the elements and determine their relative abundances in the examined samples. The SEM images of the bare carbon-paste electrode reveal a microstructure featuring layers of irregular isolated flakes of graphite powder. The SEM micrographs of Bi–Sb/CPE show that Sb–Bi nano-composites are well embedded within the carbon-paste matrix, and the electrode surface shows increased roughness and porosity, consequently increasing the electroactive surface area.

The surface of Bi–Sb/CPE is a sheet material, as evidenced by EDX analysis, and the proportions of elements therein are shown Fig. 2e. The antimony and bismuth contents in the composite obtained by *in situ* electrodeposition were also determined by the SEM-EDX technique, and elemental ratios of 0.39% and 2.43% were obtained for antimony and bismuth, respectively.

**Fig. 3:**
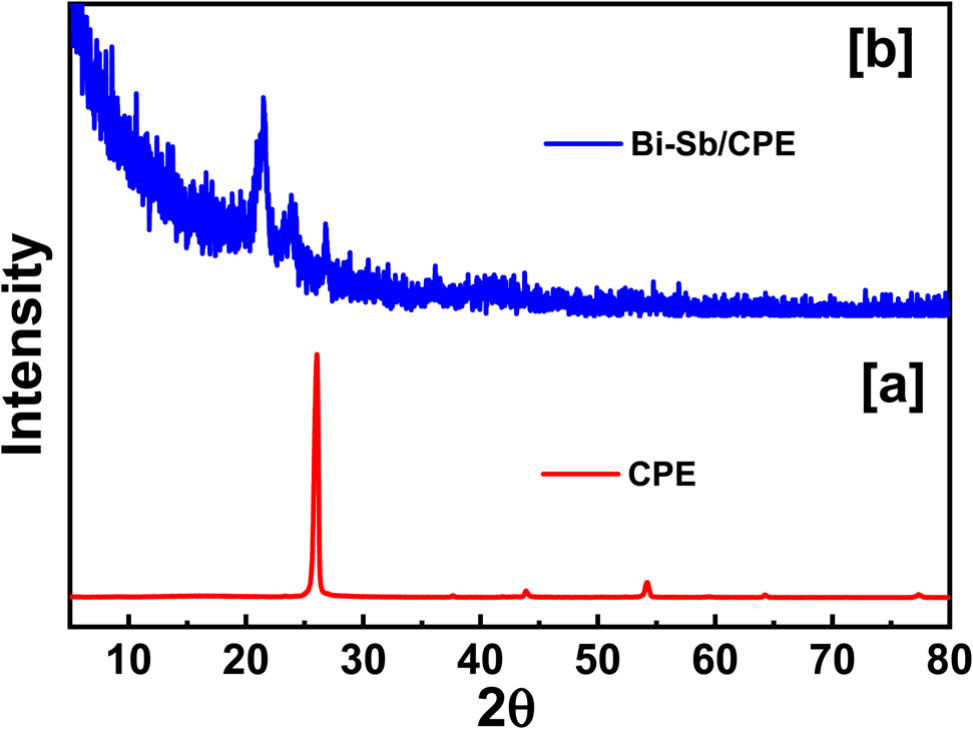
XRD patterns of electrode surfaces of bare CPE and as-prepared Bi–Sb/CPE.

Surface of both bare CPE and modified Bi–Sb/CPE were tested by XRD patterns as shown in Fig. 3. Graphite pattern appeared at 26°,^36^ due to the presence of the oxygenated groups on carbon sheets (Fig. 3a). Fig. 2b exhibited the XRD diffraction patterns of the surface of the modified Bi–Sb/CPE after the electrodeposition of Bi and Sb nanocomposite on the graphite paste. As shown in Fig. 3b, Bi nanoparticles appeared at 23.84°,^37^ and Sb nanoparticles were recorded at 21.64°, and 36.1°,^38^ while the graphite patterns seemed weak pattern at 26.97° with a small shift compared to the graphite electrode. This behavior confirmed the deposition of Bi and Sb nanocomposite on the graphite surface.

### 3.2 Electrochemical behavior and effective surface area of the Sb–Bi/CPE electrode

**Fig. 4:**
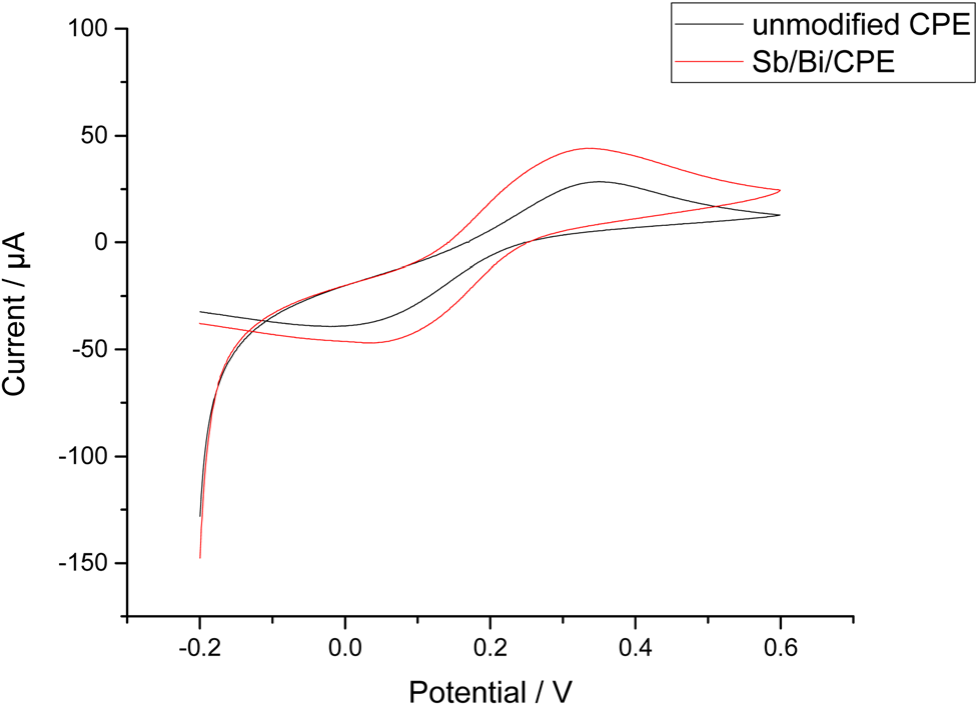
Cyclic voltammetry (CV) results for 1.0 mM K_4_[Fe(CN)_6_] in 0.1 M KCl using a scan rate of 0.1 V s^−1^ at the bare CPE and Bi–Sb/CPE.

The CV responses of the bare CPE and Bi–Sb/CPE were investigated (Fig. 4) using a 1.0 mM potassium ferrocyanide (K_4_[Fe(CN)_6_]) solution containing 0.1 M KCl. Higher oxidation and reduction peak currents are observed for the Bi–Sb/CPE than for the bare CPE, indicating better electrochemical catalytic activity and improved electron transport at the modified electrode surface.

**Fig. 5:**
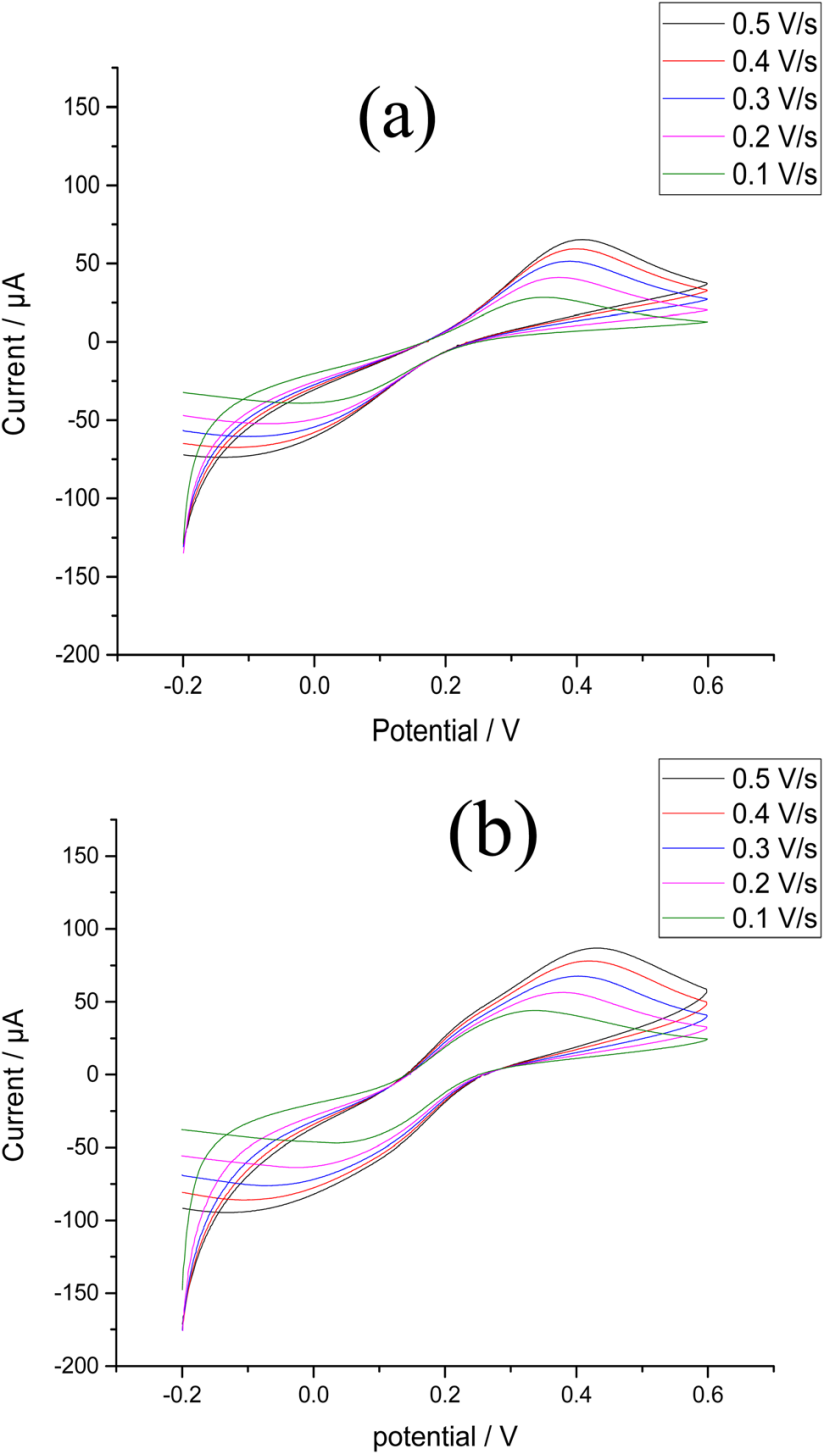
CV results for 1.0 mM K_4_[Fe(CN)_6_] in 0.1 M KCl using scan rates of 0.1–0.5 V s^−1^ at (a) the bare CPE (b) the Bi–Sb/CPE.

We can calculate the active surface areas of the bare CPE and Bi–Sb/CPE using the Randles–Sevcik equation (eqn (1)):

1 *I*_p_ = (2.69 × 10^5^)*n*^3/2^*AD*^1/2^*Cv*^1/2^

where *I*_p_ corresponds to the current (A), *n* corresponds to the number of electrons transferred during the oxidation reduction reaction (*n* = 1), *A* corresponds to the active surface area of the electrode (cm^2^), *D* corresponds to the K_4_[Fe(CN)_6_] diffusion coefficient (7.6 × 10^−6^ cm^2^ s^−1^), *C* corresponds to the K_4_[Fe(CN)_6_] concentration (mol L^−1^), and *v* corresponds to the scan rate (V s^−1^). Fig. 5a and b show the effects of scan rate on the peak currents for K_4_[Fe(CN)_6_] at the bare and modified electrodes. The slope of the relationship between the square root of the scan rate and peak current was utilized to calculate active surface area. The active surface areas of the bare CPE and the Bi–Sb/CPE are 0.052 and 0.168 cm^2^, respectively. The improvement of the active surface area supports the results obtained by SW-ASV of the target metal ions on the surfaces of the bare and modified electrodes.

### 3.3 EIS

**Fig. 6:**
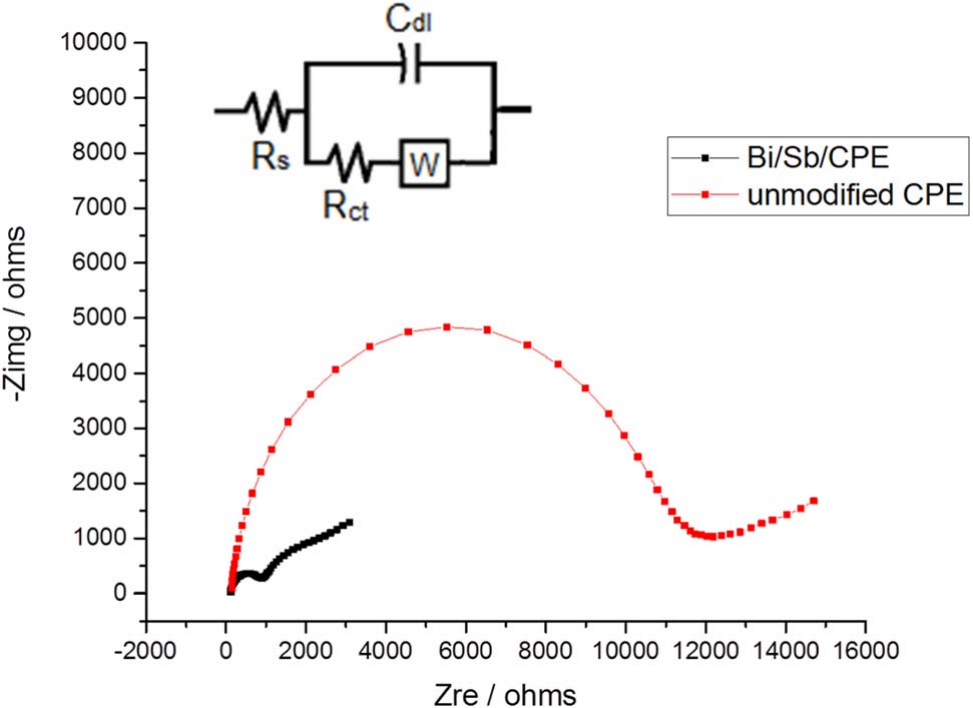
Nyquist plots for bare CPE and Bi–Sb/CPE in the presence of 1.0 mM K_4_[Fe(CN)_6_] and 0.1 M KCl at frequencies of 10 kHz to 0.1 Hz. Inset is the equivalent circuit.

The EIS technique is commonly used to study the barrier properties of electrode surfaces. The obtained curve in our study using EIS techniques called the Nyquist plot. In the high-frequency region, it is semicircle-shaped and corresponds to the electron-transfer process, while in the low-frequency region; it is linear and corresponds to the diffusion process. The semicircle diameter can be used to estimate the charge-transfer resistance (*R*_ct_), which reflects the facility of the electrode reaction. Fig. 6 shows that semicircle with a greater diameter is produced at the bare CPE, and the *R*_ct_ value for the [Fe(CN)_6_]^3−^/[Fe(CN)_6_]^4−^ oxidation reduction process was determined to be 11.446 kΩ. However, the diameter of the semicircle is considerably decreased for Bi–Sb/CPE, and *R*_ct_ was determined to be 0.9739 kΩ. This large decrease is because Bi and SbNPs greatly improve the electrical-transport properties, which facilitates the oxidation reduction process of the [Fe(CN)_6_]^3−^/[Fe(CN)_6_]^4−^ system. The results from impedance study are consistent with those obtained from CV measurements, indicating that Bi and SbNPs were successfully electrodeposited onto the bare CPE. The standard constants of heterogeneous rates for the bare CPE and Bi–Sb/CPE were calculated according to the eqn (2):

2 *k*° = *RT*/*F*^2^*R*_ct_*AC*

where *k*° corresponds to the standard heterogeneous electron-transfer rate constant (cm s^−1^), *T* corresponds to the thermodynamic temperature (298.15 K), *R* corresponds to the universal gas constant (8.314 J K^−1^ mol^−1^), *F* corresponds to the Faraday constant (96 485C mol^−1^), *R*_ct_ corresponds to the charge transfer resistance (Ω), *A* corresponds to the surface area of the electrode (cm^2^), and *C* corresponds to the [Fe(CN)_6_]^3−/4−^ solution concentration (1.0 mM). The *k*° value is an indication of the kinetic facility of a redox pair. When a system has a high *k*° value, it takes a shorter time to reach equilibrium than one with a low *k*° value, resulting in a faster equilibrium. The *k*° constants were calculated for bare CPE and Bi–Sb/CPE to be 4.47 × 10^−9^ and 1.63 × 10^−8^ cm s^−1^, respectively. As a result, the Bi–Sb/CPE has a higher *k*° and a lower *R*_ct_ than the unmodified electrode, implying a faster electron-transfer process. Moreover, the EIS for Bi–Sb/CPE was fitted by the equivalent circuit modeling of (R(C(RW))) as shown in Fig. 6 (inset). This model was built using series components; the first one is the bulk solution resistance, *R*_s_, second one the parallel combination of the double layer capacitance, *C*_dl_, charge transfer resistor, *R*_ct_, and the Warburg impedance, *W*. *R*_ct_ at the electrode surface is equal to the semicircle diameter, which can be used to describe the interface properties of the electrode.

### 3.4 SW-ASV of Zn^2+^, Cd^2+^, and Pb^2+^

**Fig. 7:**
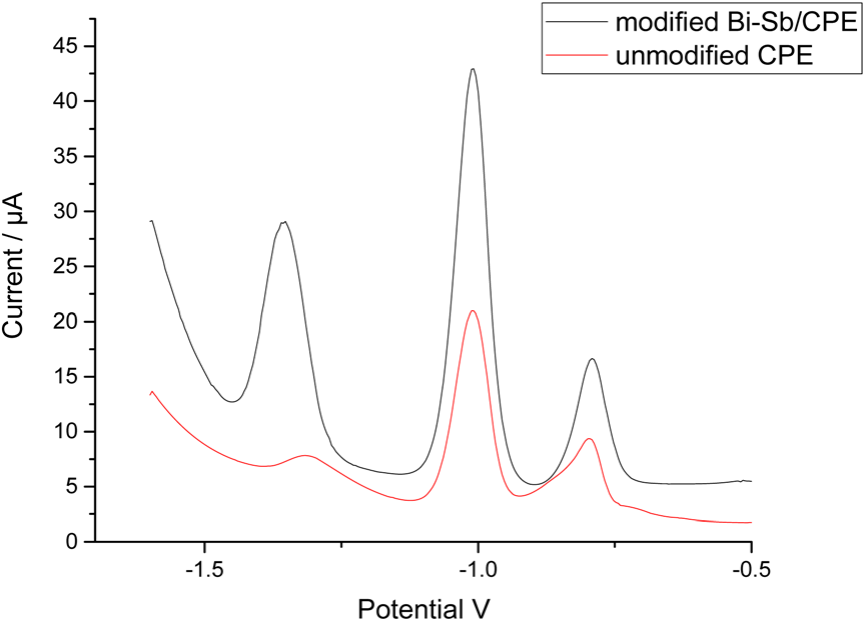
Square wave anodic stripping voltammetry (SW-ASV) results for 100 μg L^−1^ Zn^2+^, Cd^2+^, and Pb^2+^ at the bare CPE and Bi–Sb/CPE in ABS pH 5.6. (dp *E*) was −1.55 V; (dp *t*) was 180 s; frequency was 15 Hz; potential step was 6 mV; and pulse amplitude was 30 mV.

The simultaneous determination of Zn^2+^, Cd^2+^, and Pb^2+^ ions was performed at both the bare CPE and modified Bi–Sb/CPE. The SW-ASV results for 100 μg L^−1^ each of Zn^2+^, Cd^2+^, and Pb^2+^ in acetate buffer solution (ABS) pH 5.6 are shown in Fig. 7. The stripping peak current responses of Zn^2+^, Cd^2+^, and Pb^2+^ ions at the bare CPE are relatively small and appear at 1.316, 1.008, and 0.796 V, respectively. At the modified Bi–Sb/CPE, the peak separation is increased and the three peaks appear at −1.352, −1.008, and −0.792 V for Zn^2+^, Cd^2+^, and Pb^2+^, respectively. The peak current values in case of the modified Bi–Sb/CPE were 9.98, 2.19, and 2.02 times much enhanced over that of the bare CPE for Zn^2+^, Cd^2+^, and Pb^2+^, respectively. The increased peak current values could be ascribed to the Bi–Sb nanocomposite, which increases the electroactive surface area and electrical conductivity.

### 3.5 Optimization of experimental conditions

Several key factors were considered in order to achieve the best experimental conditions for SW-ASV measurement of Zn^2+^, Cd^2+^, and Pb^2+^. These parameters were the method of electrodeposition of Bi–Sb nanocomposite, electrolyte type, pH, deposition potential, and deposition time. The optimum conditions were determined based on the values of the Zn^2+^, Cd^2+^, and Pb^2+^ stripping peak currents at the same concentration (100 μg L^−1^) for each metal ion.

#### 3.5.1 Method of electrodeposition of Bi–Sb nanocomposite

**Fig. 8:**
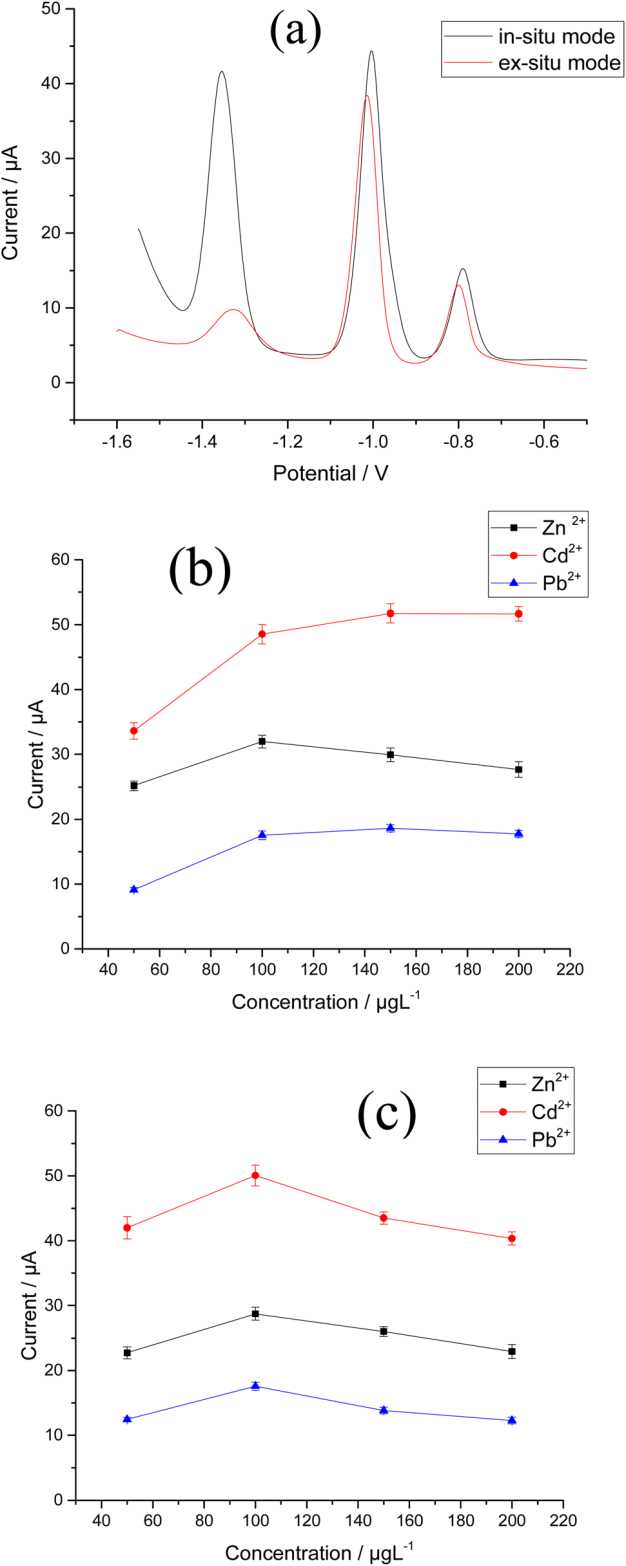
Effect of method of electrodeposition of Bi–Sb nanocomposite (a) *in situ* and *ex situ* modes (b) Bi^3+^ ion concentration (c) Sb^3+^ ion concentration on the stripping peak currents of 100 μg L^−1^ Zn^2+^, Cd^2+^ and Pb^2+^ in ABS pH 5.6, dp *E* = −1.55 V and dp *t* = 180 s at the Bi–Sb/CPE.

Zn^2+^, Cd^2+^, and Pb^2+^ were determined using Bi–Sb nanocomposite electrodeposited on the surface of a CPE in both *in situ* and *ex situ* modes (Fig. 8a). For the three target metal ions, the *in situ* electrodeposition is clearly preferable. At the Bi–Sb/CPE prepared *in situ*, the peak current values are 6.21, 1.15, and 1.13 times much enhanced than those obtained by the *ex situ* prepared electrode for Zn^2+^, Cd^2+^, and Pb^2+^, respectively. Hence, for the preparation of our modified electrode, the *in situ* mode was chosen for the following studies.

#### 3.5.2 Effect of concentration of Bi^3+^ and Sb^3+^ ions

In two separate experiments, the concentrations of either Sb^3+^ or Bi^3+^ were varied in the range 50–200 μg L^−1^ in the presence of 100 μg L^−1^ of the other metal ion. It can be seen from Fig. 8b and c that the stripping peak currents of the three determined metals are maximal when using concentration of 100 μg L^−1^ for either Bi^3+^ or Sb^3+^.

#### 3.5.3 Effect of electrolyte type and pH

**Fig. 9:**
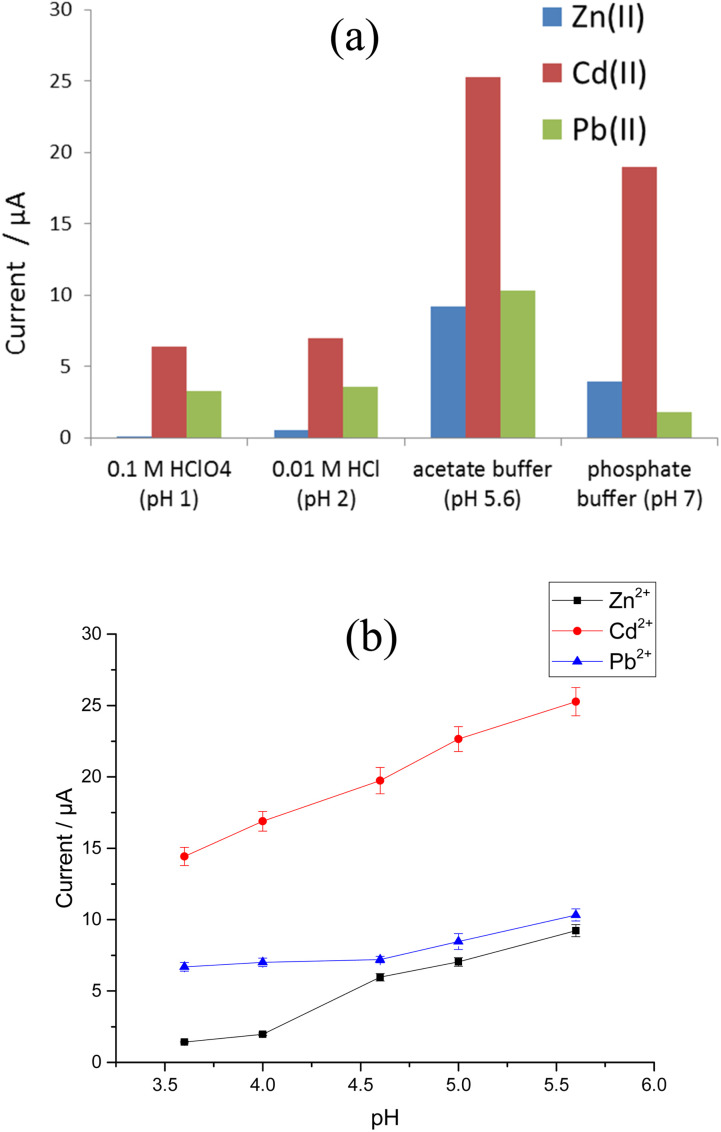
Effect of (a) type of electrolyte and (b) pH on the stripping peak currents for 100 μg L^−1^ Zn^2+^, Cd^2+^, and Pb^2+^ at the Bi–Sb/CPE.

The stripping voltammograms for 100 μg L^−1^ Zn^2+^, Cd^2+^, and Pb^2+^ were recorded at the modified Sb–Bi CPE using 0.1 M HClO_4_ (pH 1), 0.01 M HCl (pH 2), acetate buffer (pH 5.6), and phosphate buffer (pH 7) as supporting electrolytes. Fig. 9a shows the current responses for Zn^2+^, Cd^2+^, and Pb^2+^ in the four different electrolytes. Peaks corresponding to the three target metal ions are observed for all studied electrolytes, where remarkable improvements in peak current are observed for pH 5.6. Thus, the influence of pH on the stripping peak current was investigated in ABS with a pH range between 3.6 and 5.6, and the results are illustrated in Fig. 9b. The greatest Zn^2+^, Cd^2+^, and Pb^2+^ stripping peak currents are observed at pH 5.6.

#### 3.5.4 Parameters affecting SW-ASV response

Instrumental factors such as frequency, potential step, and amplitude that affect SW-ASV response were studied (Fig. S1a–c†). The frequency was investigated between 5 and 35 Hz, and 15 Hz shows the best performance. Various step-potentials between 2 and 10 mV were tested, indicating that 6 mV is optimal. The square wave amplitude was investigated between 5 and 40 mV. The optimal value was found to be 30 mV. Therefore, the optimal instrumental conditions of frequency = 15 Hz, step-potential = 6 mV, and amplitude = 30 mV were used in the rest of the study.

#### 3.5.5 Effect of accumulation conditions

**Fig. 10:**
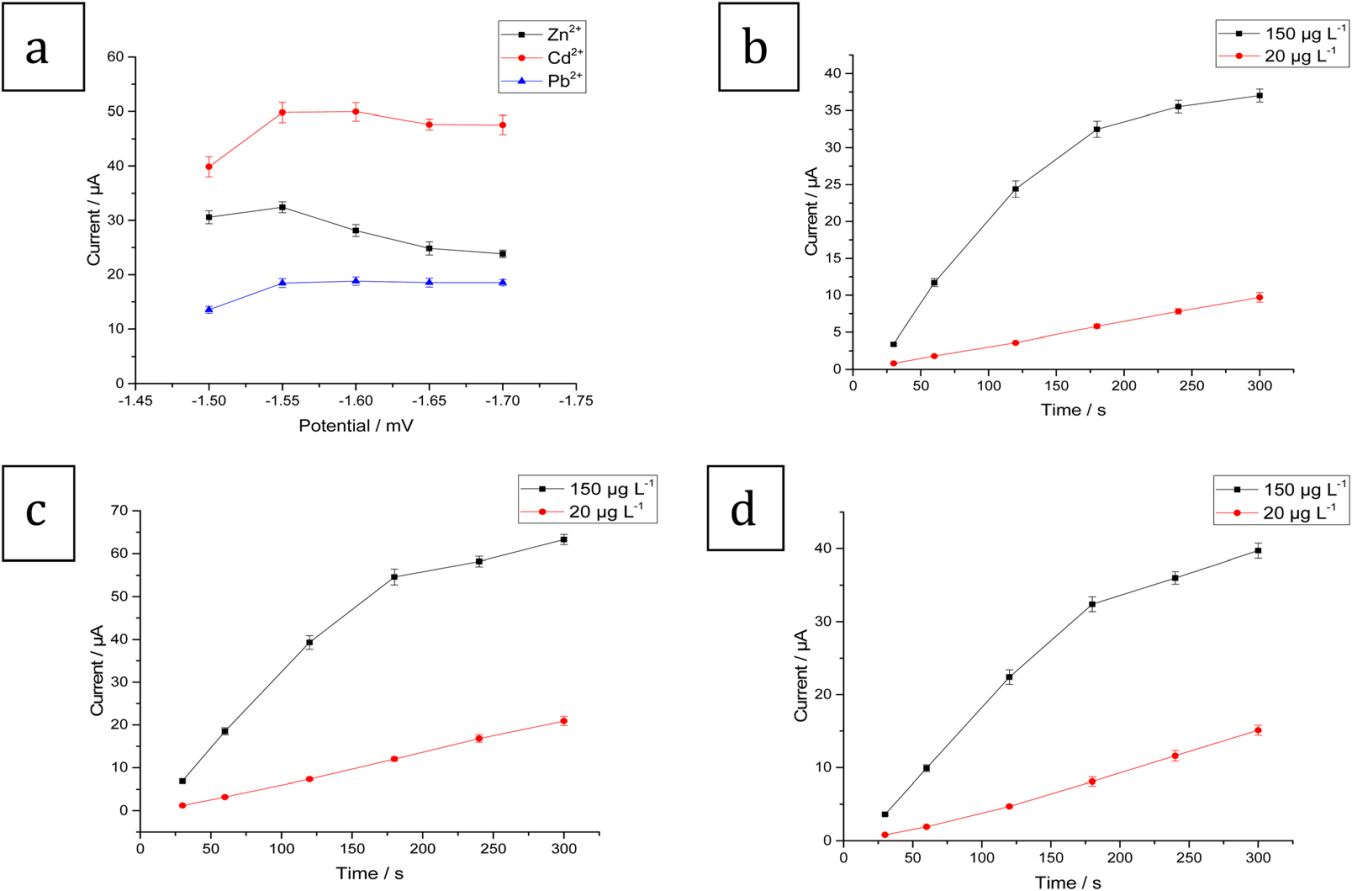
(a) Effect of accumulation potential on the stripping peak currents for 100 μg L^−1^ Zn^2+^, Cd^2+^, and Pb^2+^. Effect of accumulation time (30, 60, 120, 180, 240, and 300 s) on the stripping peak currents for 150 and 20 μg L^−1^ (b) Zn^2+^, (c) Cd^2+^, and (d) Pb^2+^ in ABS pH 5.6 and dp *E* = −1.55 V at the Bi–Sb/CPE.

The influence of deposition potential on Zn^2+^, Cd^2+^, and Pb^2+^ SW-ASV responses was investigated in the potential range −1.5 to −1.7 V. When the deposition potential shifts from −1.5 to −1.55 V, the stripping peak currents for the three metals increase (Fig. 10a). However, when the accumulation potential became more negative, the stripping peak currents decrease. The decrease in the current of the peaks can be attributed to competition with H_2_ formation. As a result, in order to achieve the highest sensitivity, −1.55 V was selected as the best deposition potential.

SW-ASVs of 20 and 150 μg L^−1^ of Zn^2+^, Cd^2+^, and Pb^2+^ were obtained under the optimal conditions after preconcentration by electrodeposition at −1.55 V for increasing preconcentration times (30 to 300 s). The peak currents for 20 μg L^−1^ Zn^2+^, Cd^2+^, and Pb^2+^ increase in a linear manner with preconcentration time over the studied period, as illustrated in Fig. 10b–d. The peak currents for 150 μg L^−1^ Zn^2+^, Cd^2+^, and Pb^2+^ show a linear rise up to 180 s. At longer preconcentration periods, the Bi–Sb/CPE surface is saturated by the deposited metals and therefore the currents of the peaks plateau. Thus, 180 s was chosen for further experiments to achieve wider concentration ranges of determination.

### 3.6 Method validation

#### 3.6.1 Linear range and detection limit

**Fig. 11:**
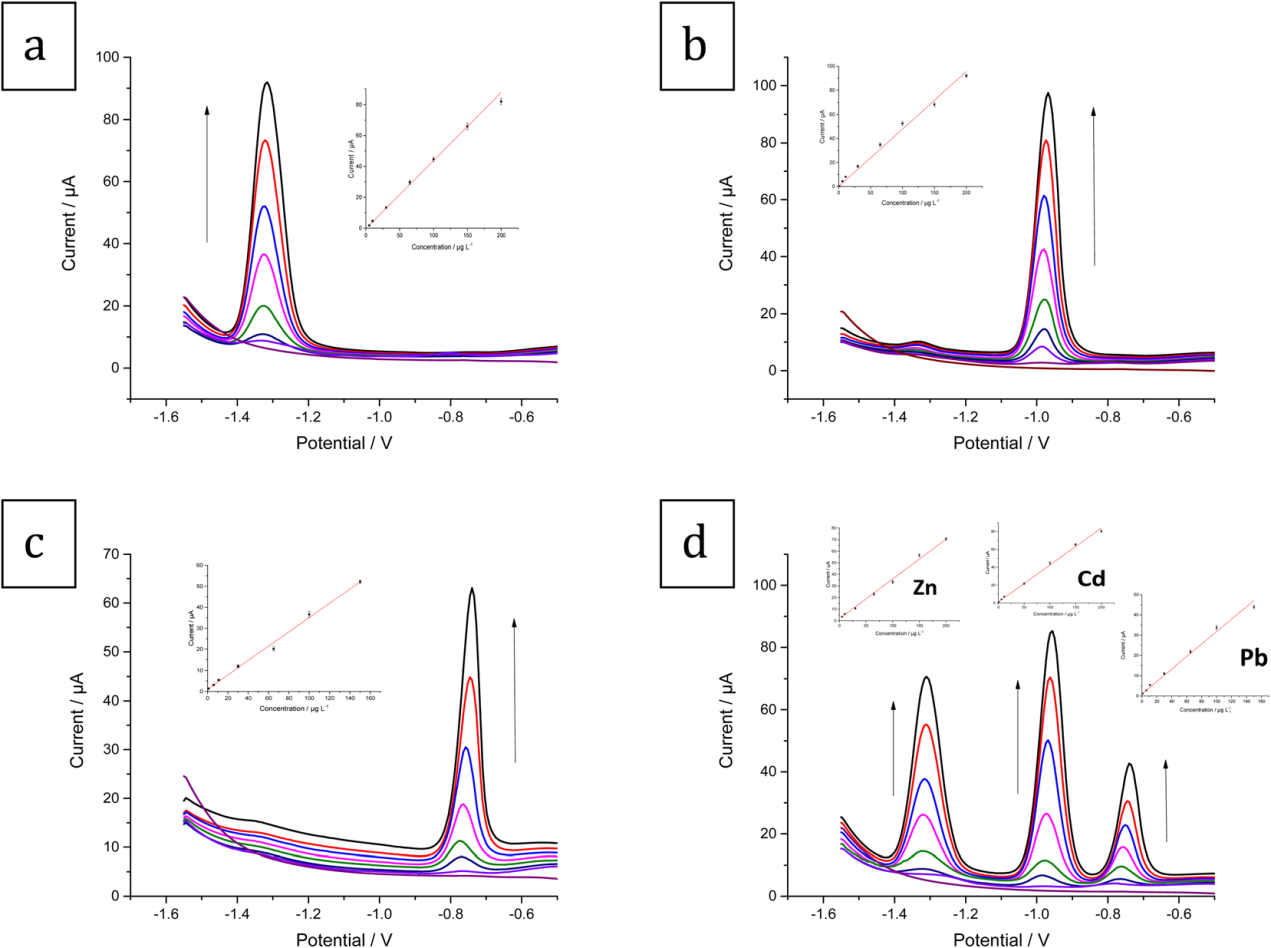
SW-ASV results and calibration for (a) Zn^2+^ (0 (blank), 5, 10, 30, 65, 100, 150, 200 μg L^−1^) (b) Cd^2+^ (0 (blank), 1, 6, 11, 30, 65, 100, 150, 200 μg L^−1^) (c) Pb^2+^ (0 (blank), 1, 6, 11, 30, 65, 100, 150 μg L^−1^) (d) the three metal ions simultaneously: Zn^2+^ (0 (blank), 5, 10, 30, 65, 100, 150, 200 μg L^−1^), Cd^2+^ (0 (blank), 1, 6, 11, 50, 100, 150, 200 μg L^−1^), and Pb^2+^ (0 (blank), 1, 6, 11, 30, 65, 100, 150 μg L^−1^) in ABS pH 5.6, dp *E* = −1.55 V and dp *t* = 180 s at the Bi–Sb/CPE.

Under the optimal experimental conditions, Zn^2+^, Cd^2+^, and Pb^2+^ were determined individually and simultaneously with the Bi–Sb/CPE using SW-ASV. The SW-ASV responses of Zn^2+^ at various concentrations are shown in Fig. 11a. In the range between 5 and 200 μg L^−1^, well-defined peaks proportional to the concentration of Zn^2+^ are observed. The sensitivity value of 0.4189 μA μg L^−1^ (*R*^2^ = 0.997) was obtained. The limit of detection (LOD) was calculated to be 1.46 μg L^−1^. The SW-ASV responses of the Sb–Bi/CPE toward Cd^2+^ over the concentration range between 1 and 200 μg L^−1^ are illustrated in Fig. 11b. The Cd^2+^ concentration and peak current exhibit a good linear relationship. The sensitivity value is 0.4523 μA L μg^−1^ (*R*^2^ = 0.995) with a LOD value of 0.27 μg L^−1^. Fig. 11c shows that the current for the Pb^2+^ peak rises in a linear manner with concentration in the range 1–150 μg L^−1^. The sensitivity value is 0.3403 μA μg L^−1^ (*R*^2^ = 0.994) with a LOD of 0.29 μg L^−1^. Therefore, Bi–Sb/CPE shows great promise as a platform for determining HMIs.

Fig. 11d shows the SW-ASV results for the measurement of Zn^2+^, Cd^2+^, and Pb^2+^ simultaneously at increasing concentrations using Bi–Sb/CPE under the optimal experimental conditions. The developed electrode was successful in determining the three target metal ions simultaneously. The voltammetric peaks are sufficiently separated, and thus simultaneous and selective detection with the Bi–Sb/CPE sensor is possible.

The calibration curve equations for the individual and simultaneous detection of the target metal ions are provided in Table 1. Comparison of the slopes of the calibration curves for each metal ion in the absence and presence of the other two target metal ions shows that Bi–Sb/CPE can be utilized for the voltammetric detection of Zn^2+^, Cd^2+^, and Pb^2+^ individually or simultaneously without any interference between the three analytes. Table 2 compares the data obtained using the proposed Bi–Sb/CPE and other different modified electrodes reported previously in the literature. As shown in Table 2, our method provides wide detection ranges and the lowest LOD values for the investigated metal ions.

**Table 1:** Analytical parameters for Zn^2+^, Cd^2+^, and Pb^2+^ detection at the Bi–Sb/CPE

	Metal ion	Regression equation: [*i* (μA)/C (μg L^−1^)]	*R* ^2^	LOD (μg L^−1^)	LOQ (μg L^−1^)
Individual determination	Zn^2+^	*i* (μA) = 0.4189 ± 0.0142C (μg L^−1^) + 1.0278 ± 0.2044	0.997	1.46	4.88
	Cd^2+^	*i* (μA) = 0.4523 ± 0.0117C (μg L^−1^) + 2.8304 ± 0.0411	0.995	0.27	0.91
	Pb^2+^	*i* (μA) = 0.3403 ± 0.0071C (μg L^−1^) + 1.0404 ± 0.0333	0.994	0.29	0.98
Simultaneous determination	Zn^2+^	*i* (μA) = 0.3505 ± 0.0112C (μg L^−1^) + 1.0457 ± 0.1724	0.995	1.48	4.92
	Cd^2+^	*i* (μA) = 0.405 ± 0.0145C (μg L^−1^) + 2.1456 ± 0.0381	0.997	0.28	0.94
	Pb^2+^	*i* (μA) = 0.2933 ± 0.0129C (μg L^−1^) + 1.9331 ± 0.0282	0.992	0.29	0.96

**Table 2:** Comparison between the prepared electrode and other related modified electrodes reported in the literature for the detection of Zn^2+^, Cd^2+^, and Pb^2+^^a^

Electrode material	Method	*t*_dep_ (s)	Linear range (μg L^−1^)			LOD (μg L^−1^)			Ref.
			Zn^2+^	Cd^2+^	Pb^2+^	Zn^2+^	Cd^2+^	Pb^2+^	
GCE-GO-BiNPs	SWASV	120	—	11.24–158.48	20.72–292.15	—	2.92	6.22	29
GC/rGO-SbNPs	SWASV	120	—	11.24–505.8	20.72–932.4	—	5.09	3.42	33
Cr-CPE	SWASV	100	80–800	10–800	10–800	25	3	3	39
D/G nano-platelets film electrode	DPASV	270	10–250	10–250	25–250	9.12	2.45	3.05	40
AgNP/Bi/Nafion	SWASV	360	5–400	0.5–400	0.1–500	5.0	0.5	0.1	41
Nafion/G/PANI nano-composite	SWASV	240	1–300	1–300	1–300	1.0	0.1	0.1	42
Bi-SPCNTE	SIA-ASV	180	12–100	2–100	2–100	11	0.8	0.2	43
Bi/poly(*p*-ABSA)	DPASV	240	1–110	1–110	1–130	0.62	0.63	0.80	44
Bi–Sb/CPE	SWASV	180	5–200	1–200	1–150	1.46	0.27	0.29	This work

a Sb-BDD: antimony nanoparticle modified boron doped diamond. D/G: diamond/graphite. AgNPs: Ag nanoparticles. G/PANI: graphene/polyaniline. SPCNTE: screen-printed carbon nanotubes electrodes. SIA-ASV: sequential injection analysis-anodic stripping voltammetry. poly(*p*-ABSA): poly(*p*-aminobenzene sulfonic acid).

#### 3.6.2 Precision and accuracy

The precision (RSD) and accuracy (% recovery) for the proposed SW-ASV method for determining Zn^2+^, Cd^2+^, and Pb^2+^ were evaluated by analyzing several standard solutions of the three metal ions. The RSD calculation was based on five replicate measurements using five freshly reproduced surfaces of the sensor, and the % recovery was obtained by applying the calibration curve method. Table of results (Table S2†) show that RSD values didn't exceed 4.13 and % recovery lied between 98.2 and 101.2%. Moreover, the peak current magnitude of 100 μg L^−1^ of each of Zn^2+^, Cd^2+^, and Pb^2+^ on the same surface of the Bi–Sb/CPE sensor was tested daily over a week. Mean recoveries of 96.11 ± 3.37, 97.53 ± 2.78 and 97.19 ± 2.55 (*n* = 7) for Zn^2+^, Cd^2+^, and Pb^2+^, respectively, were achieved. These measurements confirmed suitable repeatability, reproducibility, re-usability, and long-term stability of the modified Bi–Sb/CPE sensor for electroanalytical sensing of the target metal ions.

#### 3.6.3 Interference effect

The effects of possible interfering cations and anions present in water samples were studied before applying the modified electrode to real environmental samples analysis. Interferences from major cations (Na^+^, K^+^, Ca^2+^, and Mg^2+^), some trace metal ions (Mn^2+^, Fe^3+^, and Co^2+^) and major anions (Cl^−^, SO_4_^2−^ and HCO_3_^−^) that are typically present in real water samples on the current response of 100 μg L^−1^ of each of Zn^2+^, Cd^2+^, and Pb^2+^ were assessed (Table 3). The interference tolerance level for each interfering species is defined as the greatest concentration of interfering species that results in a ±5% variation in peak current. The obtained results demonstrate that the Bi–Sb/CPE can be considered a good sensor for detecting Zn^2+^, Cd^2+^, and Pb^2+^ in real water samples.

**Table 3:** Effect of various interfering species on the peak currents of 100 μg L^−1^ of each of Zn^2+^, Cd^2+^, and Pb^2+^

Interfering ion	Tolerance level^a^ (mg L^−1^)
Na^+^	50
K^+^	60
Ca^2+^	40
Mg^2+^	40
Fe^3+^	3
Mn^2+^	1
Co^2+^	1
Cl^−^	80
SO_4_^2−^	50
HCO_3_^−^	120

a Peak current deviation ±5%.

### 3.7 Application to real water samples

The electrochemical performance of the Bi–Sb/CPE sensor was evaluated with a variety of real water samples, including tap water, bottled water, underground water, and treated waste water. Since Zn^2+^, Cd^2+^, and Pb^2+^ were not found in the first three water samples, our study was continued by spiking the samples with three different concentrations (15, 75, and 150 μg L^−1^) of each metal ion. The three metal ions were all found in the treated waste water and were measured using the calibration curve method without spiking. Results are tabulated in Table 4 and it shows that RSD values didn't exceed 4.54 and % recovery lied between 95.2 and 103.7%. The results demonstrate that the developed Bi–Sb/CPE sensor exhibits good accuracy for Zn^2+^, Cd^2+^, and Pb^2+^ detection in environmental water samples.

**Table 4:** Detection of Zn^2+^, Cd^2+^, and Pb^2+^ in different samples of real water using the proposed method

Type of water	Metal ions	Spiked (μg L^−1^)	Found (μg L^−1^)	% recovery	RSD
Tap water	Zn	15	14.60	97.3	3.55
		75	71.85	95.8	4.23
		150	147.15	98.1	2.34
	Cd	15	14.43	96.2	1.26
		75	73.58	98.1	2.47
		150	145.20	96.8	2.75
	Pb	15	14.46	96.4	4.34
		75	71.78	95.7	4.73
		150	147.75	98.5	3.34
Bottled water	Zn	15	14.58	97.2	2.44
		75	76.73	102.3	4.27
		150	147.15	98.1	2.93
	Cd	15	15.32	102.1	3.38
		75	76.05	101.4	1.32
		150	155.25	103.5	2.43
	Pb	15	15.56	103.7	4.29
		75	76.35	101.8	3.42
		150	154.80	103.2	3.38
Underground water	Zn	15	14.36	95.7	3.47
		75	72.60	96.8	1.66
		150	146.40	97.6	2.64
	Cd	15	14.13	94.2	4.32
		75	73.73	98.3	2.97
		150	146.25	97.5	3.54
	Pb	15	14.45	96.3	2.38
		75	71.40	95.2	4.54
		150	147.30	98.2	2.87
Treated waste water	Zn	—	6.11	—	2.44
	Cd	—	4.18	—	3.69
	Pb	—	5.25	—	2.95

## 4 Conclusions

A simple and efficient CPE modified with a Bi–Sb nanocomposite was successfully fabricated for stripping analysis of Zn^2+^, Cd^2+^, and Pb^2+^ metal ions. The suggested sensor demonstrates good analytical performance under optimal conditions, with LOD values of 1.46, 0.27, and 0.29 μg L^−1^ for simultaneous determination of Zn^2+^, Cd^2+^, and Pb^2+^, respectively. The described method can be effectively utilized to determine the three metal ions in real water samples without significant interference. Future projection and scope will be trying to apply the developed Bi–Sb/CPE sensor for trace determination of some biologically-active organic compounds such as drugs and pesticides.

## Author contributions

E. A. Shalaby: investigation, data curation, methodology. A. M. Beltagi: conceptualization, supervision, methodology, writing – review & editing. A. A. Hathoot: supervision, methodology. M. Abdel Azzem: conceptualization, supervision, methodology, writing – review & editing.

## Conflicts of interest

There are no conflicts to declare.

## Supplementary Material

RA-013-D3RA00168G-s001
