# Supplementary material for: Simultaneous voltammetric sensing of Zn2+, Cd2+, and Pb2+ using an electrodeposited Bi–Sb nanocomposite modified carbon paste electrode
Source: RSC Adv. 2023 Mar 2;13(11):7118–28. doi: 10.1039/d3ra00168g (PMC9978880; doi:10.1039/d3ra00168g)
Supplement: RA-013-D3RA00168G-s001 [file RA-013-D3RA00168G-s001.pdf]

Electronic Supplementary Material (ESI) for RSC Advances.
This journal is © The Royal Society of Chemistry 2023

The supplementary information document for the paper entitled:

Simultaneous voltametric sensing of Zn^{2+}, Cd^{2+}, and Pb^{2+} using an electrodeposited Bi–Sb nanocomposite modified carbon paste electrode

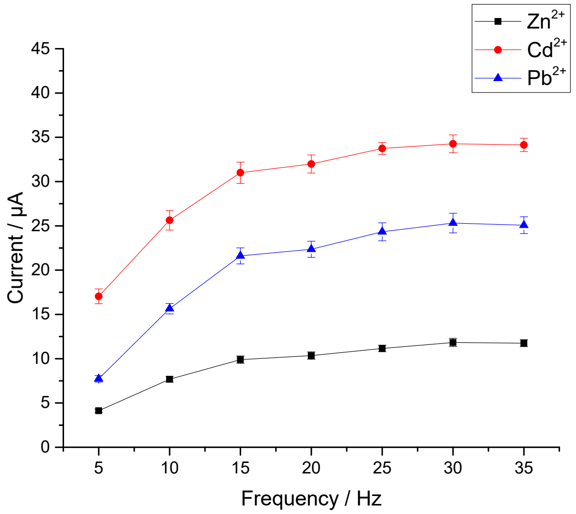

(a)

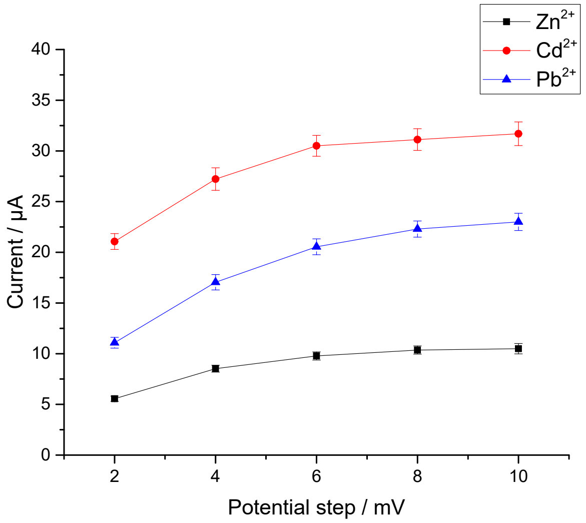

(b)

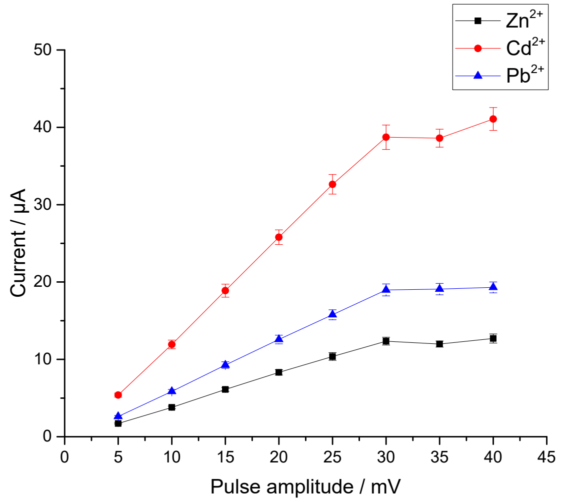

(c)

Fig. S1. Effect of (a) frequency, (b) potential step, and (c) pulse amplitude on the stripping peak currentsfor100 µgL^{-1} Zn^{2+}, Cd^{2+}, and Pb^{2+} in ABS (pH 5.6) at the Bi–Sb/CPE.

Sensitivity, LOD and LOQ calculation

The limit of detection (LOD) and the limit of quantification (LOQ) were calculated by the following equations:

LOD = 3 SD/S

LOQ = 10 SD/S

where (SD) is the standard deviation of the intercept of the calibration curve equation (3 replicates) and (S) is the sensitivity of the method (equal to the slope of calibration curve).

Table S2. Precision and accuracy of the described SW–ASV method for simultaneous determination of Zn^{2+}, Cd^{2+}, and Pb^{2+}

Metal ions	Spiked (µg L^{-1})	Found (µg L^{-1})	% recovery	RSD (n=5)
Zn	15	14.75	98.3	2.35
	75	74.63	99.5	2.94

	150	148.05	98.7	4.13
Cd	15	15.06	100.4	1.54
	75	74.48	99.31	3.27
	150	151.8	101.2	3.63
Pb	15	14.73	98.2	2.62
	75	73.8	98.4	3.44
	150	150.6	100.4	2.89
